# Supplementary material for: Sensitized piRNA reporter identifies multiple RNA processing factors involved in piRNA-mediated gene silencing
Source: bioRxiv. 2023 Jan 22:2023.01.22.525052. Preprint. [Version 1] doi: 10.1101/2023.01.22.525052 (PMC9882300; doi:10.1101/2023.01.22.525052)
Supplement: Supplement 1 [file media-1.pdf]

Title

Sensitized piRNA reporter identifies multiple RNA processing factors involved in piRNA-mediated gene silencing

Author List

Jordan Brown[1], Donglei Zhang[1], Wenjun Chen[1, 2], and Heng-Chi Lee[1*]

Affiliations

1 Department of Molecular Genetics and Cell Biology, University of Chicago, Chicago, IL 60637, USA

2 Present address: Department of Laboratory Medicine, Third Affiliated Hospital of Sun Yat-Sen University, Guangzhou, China

*Corresponding author: Heng-Chi Lee (hengchilee@uchicago.edu)

This supplementary information includes:

Figure S1. piRNA reporter screening strategy.

Figure S2. Loss of piRNAs following *snpc-4*, *ints-1*, and *dic-1* knockdown.

Figure S3. NPP-1/6/7/9 and CSR-1 pathway disruption activates the sensitized piRNA reporter.

Figure S4. HRDE-1 does not share NRDE-3's nuclear localization signal.

Supplementary Table 1. Full list of candidates screened using piRNA reporter.

Figure S1. piRNA reporter screening strategy.

A) A model depicts the cellular context of biogenesis and effector steps in piRNA silencing.

Figure S2. Loss of piRNAs following *snpc-4*, *ints-1*, and *dic-1* knockdown.

A) Scatterplots show the expression of mature type I piRNAs in the indicated RNAi knockdowns.

B) Scatterplots show the expression of type I piRNA precursors in the indicated RNAi knockdowns.

C) A histogram shows the distribution of type I and type II piRNA precursor sequence lengths in cap-enriched sequencing libraries from *ints-1* and empty vector RNAi treated worms.

D) A model shows the proposed action of the Integrator complex at piRNA loci.

Figure S3. NPP-1/6/7/9 and CSR-1 pathway disruption activates the sensitized piRNA reporter.

A) GFP expression in type II piRNA reporter in indicated RNAi knockdowns. Asterisks indicate significant reporter activation, see Methods for details.

B) GFP expression in type I piRNA reporter in indicated RNAi knockdowns. Asterisks indicate significant reporter activation, see Methods for details.

C) Percentage of worms with dispersed GFP::CSR-1 (top) or GFP::PRG-1 (bottom) granules in the indicated RNAi knockdowns. Asterisks indicate $p < 0.05$ in one-tailed Student’s t-test.

D) Percentage of germline nuclei with nucleoli that contain NPP-7 following the indicated RNAi knockdowns. Representative fluorescent micrographs show mCherry::NPP-7 localization from indicated treatments. Treatments that also activate the piRNA reported are marked with an asterisk.

Figure S4. HRDE-1 does not share NRDE-3's nuclear localization signal.

A) GFP expression in type II piRNA reporter in indicated RNAi knockdowns.

B) A selection from the Smith-Waterman local alignment of HRDE-1 and NRDE-3 amino acid sequences that includes the NRDE-3 nuclear localization signal.

Supplementary Table 1. Full list of candidates screened using piRNA reporter.

B) A model depicts the cellular context of biogenesis and effector steps in piRNA silencing.

Figure S1

A

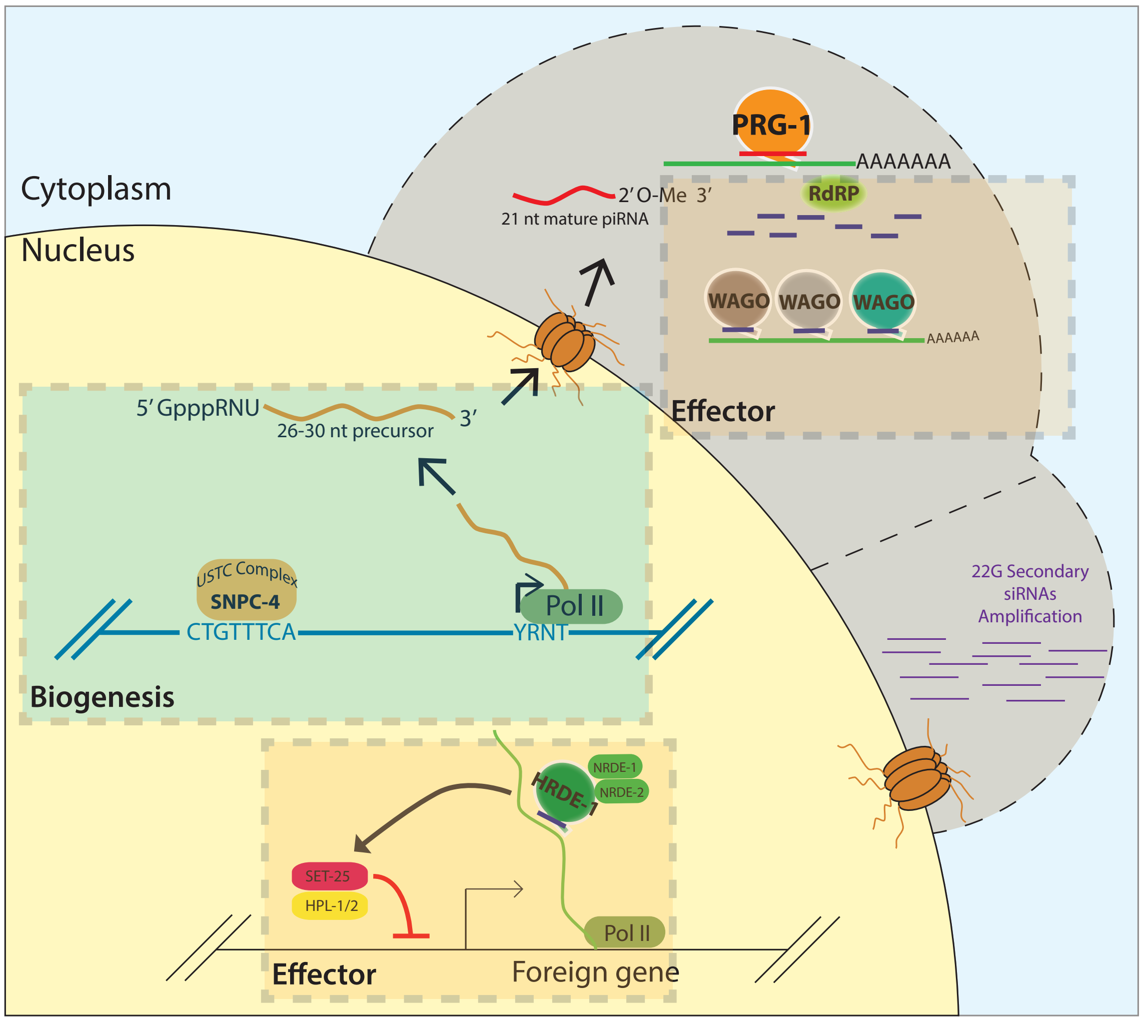

Cytoplasm
Nucleus
PRG-1
AAAAAAA
2′O-Me 3′
21 nt mature piRNA
RdRP
WAGO
WAGO
WAGO
AAAAAA
Effector
5′GpppRNU
26-30 nt precursor
3′
USTC Complex
SNPC-4
CTGTTTCA
Pol II
YRNT
Biogenesis
22G Secondary
siRNAs
Amplification
HRDE-1
NRDE-1
NRDE-2
SET-25
HPL-1/2
Pol II
Effector
Foreign gene

Figure S2

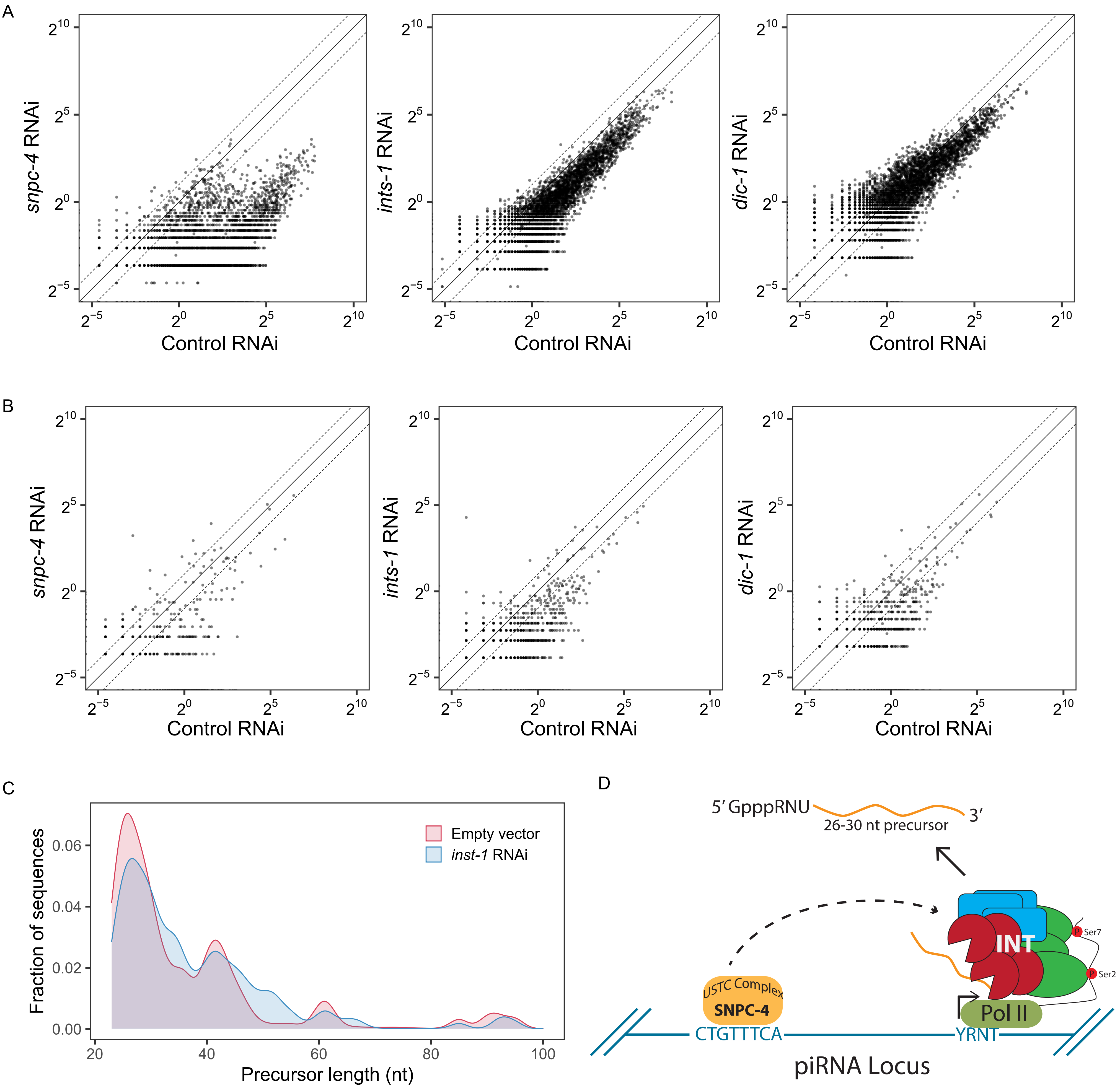

A
snpc-4 RNAi
ints-1 RNAi
dic-1 RNAi
Control RNAi
B
snpc-4 RNAi
ints-1 RNAi
dic-1 RNAi
Control RNAi
C
Fraction of sequences
Precursor length (nt)
Empty vector
inst-1 RNAi
D
5′ GpppRNU
26-30 nt precursor
3′
INT
Ser7
Ser2
USTC Complex
SNPC-4
Pol II
CTGTTTCA
YRNT
piRNA Locus

Figure S3

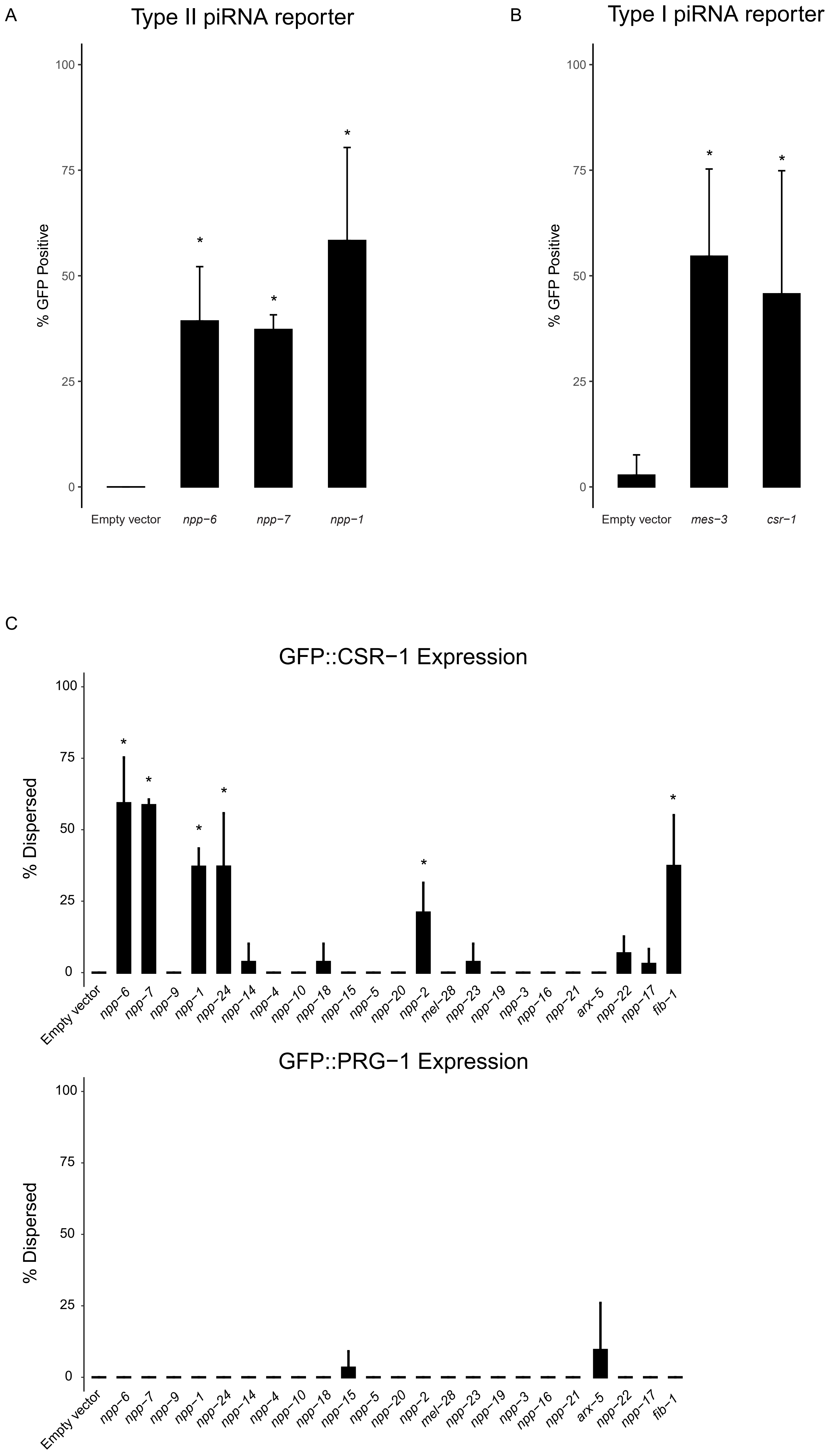
A
Type II piRNA reporter
% GFP Positive
0
25
50
75
100
Empty vector
npp−6
npp−7
npp−1
*
B
Type I piRNA reporter
% GFP Positive
Empty vector
mes−3
csr−1
C
GFP::CSR−1 Expression
% Dispersed
Empty vector
npp−6
npp−7
npp−9
npp−1
npp−24
npp−14
npp−4
npp−10
npp−18
npp−15
npp−5
npp−20
npp−2
mel−28
npp−23
npp−19
npp−3
npp−16
npp−21
arx−5
npp−22
npp−17
fib−1
GFP::PRG−1 Expression
% Dispersed

Figure S3, continued

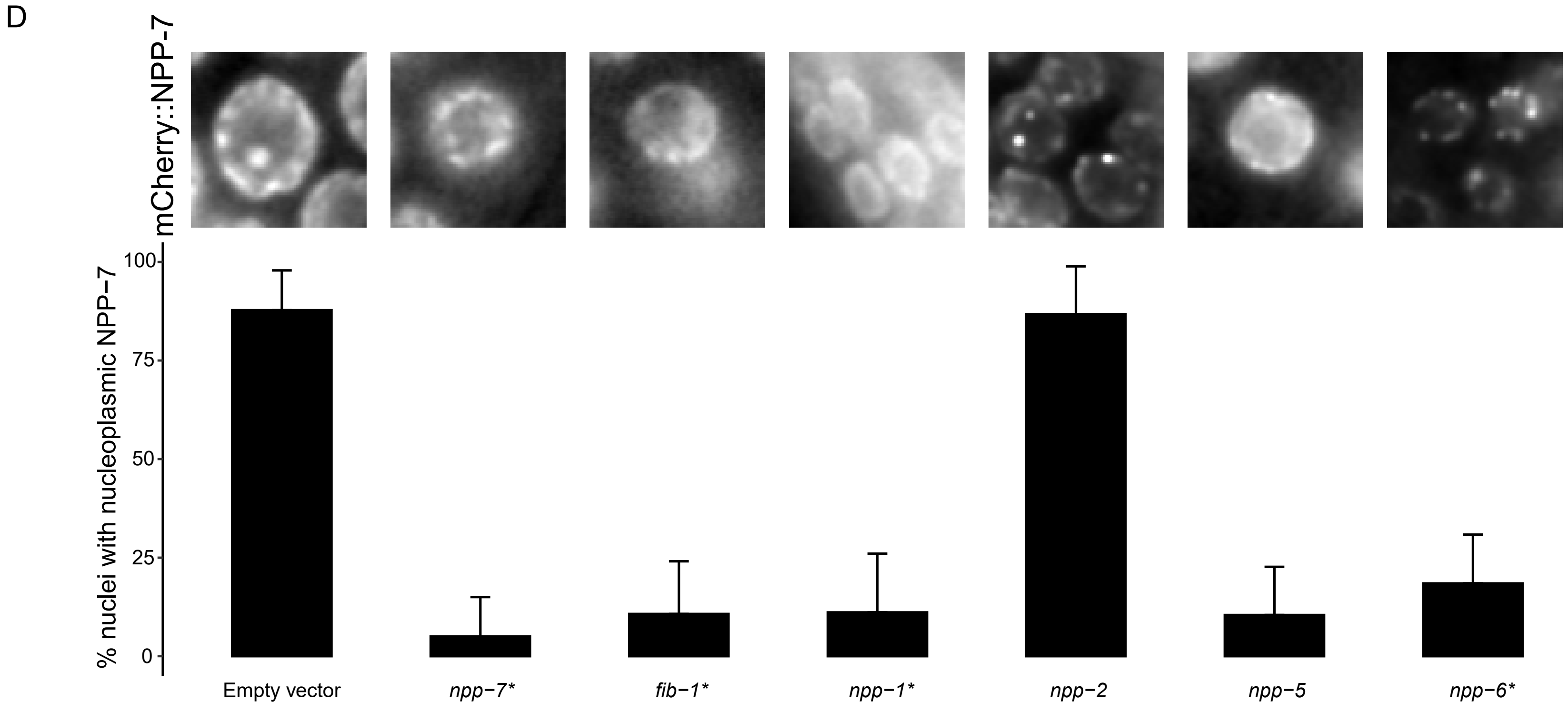

* = also activated piRNA reporter

Figure S4

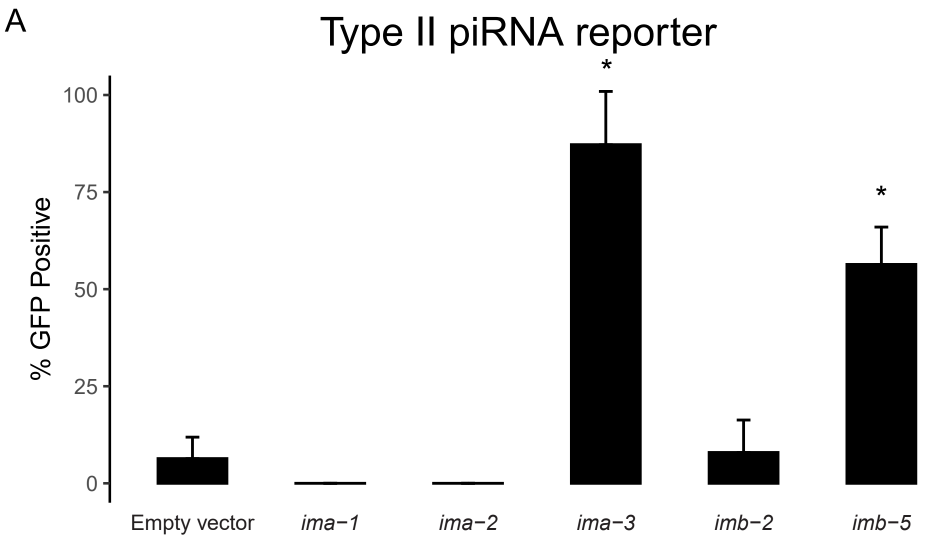
A
Type II piRNA reporter
% GFP Positive
100
75
50
25
0
*
*
Empty vector
ima−1
ima−2
ima−3
imb−2
imb−5

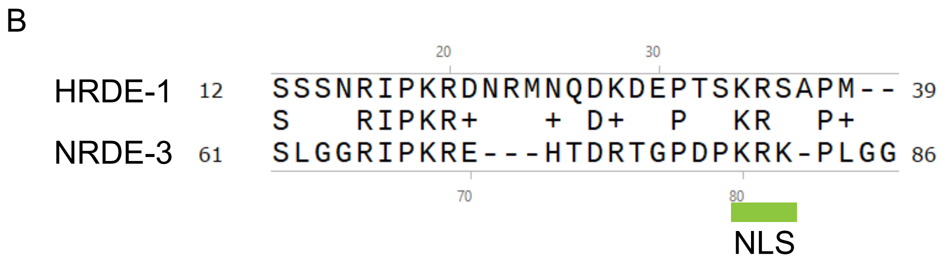
B
20
30
HRDE-1 12 SSSNRIPKRDNRMNQDKDEPTSKRSAPM-- 39
S RIPKR+ + D+ P KR P+
NRDE-3 61 SLGGRIPKRE---HTDRTGPDPKRK-PLGG 86
70
80
NLS
